# The Legacy of Parental Obesity: Mechanisms of Non-Genetic Transmission and Reversibility

**DOI:** 10.3390/biomedicines10102461

**Published:** 2022-10-01

**Authors:** Gemma Comas-Armangue, Lela Makharadze, Melisa Gomez-Velazquez, Raffaele Teperino

**Affiliations:** 1 German Research Center for Environmental Health Neuherberg, Institute of Experimental Genetics, Helmholtz Zentrum München, 85764 Neuherberg, Germany; 2 German Center for Diabetes Research (DZD) Neuherberg, 85764 Neuherberg, Germany

**Keywords:** obesity, HFD, epigenetics, DOHaD, POHaD, non-genetic inheritance, intergenerational inheritance, reversibility

## Abstract

While a dramatic increase in obesity and related comorbidities is being witnessed, the underlying mechanisms of their spread remain unresolved. Epigenetic and other non-genetic mechanisms tend to be prominent candidates involved in the establishment and transmission of obesity and associated metabolic disorders to offspring. Here, we review recent findings addressing those candidates, in the context of maternal and paternal influences, and discuss the effectiveness of preventive measures.

## 1. Introduction

Obesity is a medical condition that increases the risk of developing type 2 diabetes (T2D), cardiovascular diseases, and several types of cancer, among other pathologies [1]. It has a complex etiology, involving genetics, the environment, and the crosstalk between them. This complexity became more evident when genome-wide association studies (GWAS) began to detect more genetic variants associated with obesity, such as those in fat mass and obesity-associated (*FTO*) or melanocortin 4 receptor (*MC4R*) genes, among many others [2,3,4,5,6]. However, genetic variants alone are unable to explain the rapid spread of obesity in the population and the inherited individual susceptibility to metabolic disease development [7,8]. On the other hand, environmental changes in modern society, including poor quality diets, sedentary lifestyles [9,10], and changed working habits [11,12] tend to explain the recent obesity epidemic. Current obesity-related studies have focused on diet-induced weight gain, because the rise in obesity prevalence in recent decades is strongly associated with the composition of modern diets [13,14,15,16,17].

Interestingly, diets not only lead to a self-directed impact; evidence suggests that parental diets can also affect the health of future generations [18,19,20,21]. This is well illustrated in humans by two diet-related conditions: historical famines and modern diet-associated obesity. During the periconceptional period and/or pregnancy, both food-related events have been shown to result in offspring obesity, T2D, and metabolic phenotypes, such as hyperglycemia and cardiovascular diseases [18,22]. Apparently unrelated phenotypes, such as schizophrenia or proliferative diabetic retinopathy in association with famine [19,20], or cognitive impairment in association with obesity [21], have also been observed. Overall, although such dietary challenges differ with the amount of food that is available, food-related events during both the periconceptional period and/or pregnancy made it evident that parental malnutrition has detrimental effects on the metabolic health of offspring.

Considering the duration of direct in utero or postnatal influences, the role of a maternal diet has been largely acknowledged and extensively addressed within the scope of the developmental origins of health and disease (DOHaD), which is a study of the consequences of maternal early-life influences on offspring during pregnancy and lactation. During these periods, diseases can be developmentally programmed and result in long-term adaptive responses and adult phenotypes [23]. Paternal contributions, however, have only recently gained increased attention in this context. Associations between the increase in the prevalence of male obesity and infertility [24], as well as observed metabolic and cardiovascular negative outcomes in the offspring of obese fathers [25], have prompted a refinement in considering the paternal effects on the metabolic health of offspring. In fact, the recently coined concept of paternal origin of health and disease (POHaD) emphasizes the emerging role of the impact of paternal preconception health and paternal inheritance on offspring health, including diet-induced obesity [26].

The parental environmental information that is passed on to the following generation is mainly encrypted in the germ cells, putatively in the germ cell epigenome [27]. First coined by C. H. Waddington in 1942, the epigenome was understood as the interaction between the genotype and the phenotype that allows for cell differentiation and the canalization of the terminally differentiated state [28]. This epigenetic information is transmitted from the mother cell to the daughter cell, a process that is known as epigenetic inheritance. Twenty years later, R. Holliday introduced the idea that the epigenetic modifications could be transmitted through generations, a concept that is now known as inter- or transgenerational epigenetic inheritance, depending on the number of generations affected [29]. Contemporary researchers now define epigenetics as “the study of changes in gene function that are mitotically and/or meiotically heritable and that do not entail a change in DNA sequence” [30].

Epigenetic changes, which are also referred to as epimutations, can be triggered by various environmental stimuli. They are subject to forward–backward dynamics and occur at a more frequent rate than spontaneous genetic mutations, allowing an organism to adapt faster to the environment [31,32]. Epimutations could be transferred from one generation to the next to confer an advantage for evolutionary adaptation and fitness, but they may increase susceptibility to “environmental diseases” (such as obesity and diabetes) when the ancestral environment (e.g., famine) is different from the offspring environment (e.g., feast). This idea has been adopted in the context of obesity and is referred to as metabolic thriftiness [33]. Originally, the concept implied that parental exposure to food shortage can initiate better adaptation to the environment by promoting efficient energy storage, and therefore a higher chance of survival [33]. However, when the environment imposes the opposite situation—excessive nutrition—this feature can result in obesity and associated T2D.

Mechanistically, metabolic thriftiness and the associated evolutionary adaptation were originally attributed to so-called “thrifty genes”, which were supposedly selected during evolution to promote the efficient handling of energy [33]. Because the speed of genetic adaptation and variation cannot explain the current pace of the obesity epidemic [7,8], metabolic thriftiness has lately been attributed to epimutation (the so-called thrifty epigenotype), which—by overcoming slow genetic evolution—could be responsible for the establishment and transmission of the thrifty phenotype across generations [33,34,35].

When referring to the epigenetic inheritance across generations, important distinctions are drawn with respect to specificities of parent–offspring interactions. In fact, maternal environmental exposures that generate epigenetic changes in offspring (F1) do not represent epigenetic inheritance per se. Instead, these epigenetic modifications are the epigenetic response of the embryo/offspring to direct exposure to the maternal environment in the uterus, which is more accurately described as developmental programming. When such responses persist in further generations in the absence of the environmental insult, it is assumed that the original information was stored in the primordial germ cells, persisted throughout development, and led to epigenetic inheritance. These responses can reach one or more generations, a process that is called inter- (F2) or transgenerational (F3 and further) epigenetic inheritance, respectively. On the other hand, as father–offspring interaction and information transfer are indirect (i.e., the father does not directly interact with the offspring during development) and limited in space (i.e., interaction only in the maternal tract) and time (i.e., conception), paternal intergenerational epigenetic inheritance applies to F1, while paternal transgenerational epigenetic inheritance applies to F2 and further generations.

To mimic dietary obesity in humans, diet-induced obesity has been modelled in rodents. Parental obesogenic diets that are rich in fat and/or sugar have confirmed the transmission of metabolic phenotypes inter- and/or transgenerationally in such experimental models [36,37]. Current research addresses how an obesogenic diet influences the parental epigenome, how epimutations are transferred to the next generation, and how these epigenetic changes affect offspring health. Focusing on the effects of parental diet-induced obesity in animal models—as a result of a high-fat diet (HFD) or a high-fat, high-sugar “western diet” (WD)—and in humans, the aim of this review is to provide an overview of the likely underlying molecular mechanisms behind the intergenerational consequence of parental obesity and the current approaches to its reversibility.

## 2. Maternal Obesity Affects Offspring Health

Mothers have a close interaction with offspring during pregnancy and early life. Research in humans and mice has made us aware that maternal obesity is directly related to poor offspring health [38], not only as a consequence of obesity-triggered pregnancy complications [39], but also as a result of epigenetic changes that are stable throughout generations. Indeed, several studies have shown that maternal obesity or increased fat intake induce transcriptional reprogramming [40], histone modifications [41,42], and DNA methylation changes in offspring [43,44,45,46]. These epigenetic modifications have been reported in human and animal fetal tissues [41], placenta [46], cord blood [43,44], newborns [45], and adults [42].

The interaction between the mother and the offspring can be divided into three susceptibility time windows: (i) before pregnancy (preconception) [44,46,47,48], (ii) during gestation [46], and (iii) during the postnatal period (lactation) [42,49]. Exposures during each of these susceptibility windows affect distinct offspring developmental stages and involve a specific mediator of information transfer: the oocyte during preconception; mainly the placenta during pregnancy; and breast milk postnatally. In addition, there are other less-studied putative mediators, such as vaginal and breast milk microbiota [50,51,52,53,54], the maternal immune system [55], and early postnatal care [56].

### 2.1. Preconceptional Maternal Obesity: Effects in the Oocyte

Oogenesis in humans starts while the female embryo is in the maternal uterus, continues after puberty, and completes development if fertilization takes place. Throughout its lifetime, all these cells are permanently exposed to maternal environmental conditions. There is growing information about the developmental epigenetic changes that occur during oogenesis and about their inheritance [57]. Although there is limited information regarding epigenetic changes in the event of environmental insults, it is well known that before pregnancy, oocyte quality can be affected by obesity, resulting in poor fertility [58].

It has been suggested that maternal preconceptional feeding with a HFD is sufficient to induce epigenetic changes in oocytes and transmit metabolic phenotypes to offspring. Some studies in mice have addressed such changes globally. For example, oocytes from HFD-fed mothers present a decrease in 5-methylcytosine (5mC) levels and an increase in 5-hydroxymethylcytosine (5hmC) levels [59,60]. The same studies also found an increase of histone H3 lysine 4 trimethylation (H3K4me3) and histone H3 lysine 9 trimethylation (H3K9me3) [60]. Some studies in mice have also demonstrated that a HFD causes epigenetic changes, such as altered methylation of the leptin promoter [61]. Although the specific epigenetic changes present in F1 are unknown, there is evidence from non-HFD-related studies that suggests that the epigenetic changes in the oocyte may be present in offspring. For example, the same alterations in DNA methylation of the insulin-like growth factor 2 (*Igf2*) gene found in rat oocytes that are exposed to an excess of androgens were also detected in F1 β-cells [62].

There is a potential mechanism to explain how a HFD changes the epigenome in the oocyte, which can be transmitted to the following generation. A HFD reduces the levels of Stella protein in oocytes. This protein is a maternal factor that is essential for early development and has a role in global epigenetic remodeling after fertilization. Stella downregulation in murine oocytes that originate from HFD-fed females is coupled with compromised development and reduced 5mC levels in the maternal pronucleus in the zygote. The latter prevents epigenetic asymmetry in the early embryo. Overexpressing Stella in these oocytes recovers, to a certain extent, 5mC levels in the maternal pronucleus in the zygote and in specific methylated regions [63]. These results suggest, at least in part, that HFD effects could be mediated by Stella protein.

Although maternal intergenerational effects are the most studied in mammals, detailed information on diet-induced epigenome reprogramming in oocytes is limited, most likely by the fact that oocytes are difficult to obtain in big numbers in physiological settings (e.g., without superovulation). New single-cell technologies, however, have already provided detailed DNA methylation and chromatin accessibility in mice and humans during oogenesis [64,65], promising to fill this gap and thereby enabling us to tackle questions raised by the current data: What are the genes on which epigenetic marks act upon? Can the same epigenetic mark in the oocyte be found in the adult offspring tissues? What could be the reason for a discrepancy between oocyte and F1 tissues?

### 2.2. Maternal Obesity during Pregnancy: Effects in the Placenta

During pregnancy, the placenta acts as the interface between the mother and the fetus. Its roles include fetal nutrient supply, removal of fetal metabolic waste, and hormone production [66]. Obesity during pregnancy can affect placental physiology and structure. Indeed, some studies suggest that mothers with high body mass indices (BMI) display heavier placentas [67], similar to HFD-fed mice [68]. The alterations in a placenta due to obesity can have a negative impact on offspring, as recent studies indicate that a HFD can affect placental function, leading to fetal growth restriction [69,70]. Little is known about how the maternal diet and/or obesity can alter placental function and/or structure. A study of mice showed that a HFD causes hypomethylation, in comparison with a control diet, but this difference was only significant in female placentas [68]. Another study, of humans, showed that maternal obesity can negatively affect the placenta by altering the DNA methylation of genes related to the biological processes of sterol regulatory element-binding protein (SREBP) signaling and phospholipid transport, among other processes [71]. SREBPs are membrane-bound transcription factors that control lipid synthesis and uptake [72]. The authors of that study also found that maternal saturated-fat intake altered the methylation levels of genes that are involved in biological processes related to chromatin remodeling, insulin-like growth factor (IGF) receptor signaling, phosphoinositid-3-kinase (PI3-kinase) signaling, and nitric oxide synthase [71]. Researchers have proposed that it is necessary to determine if the DNA methylation dysregulation affects placental nutrient transport, which could explain the fetal growth restriction [71]. Gabory et al. observed that a HFD downregulated the histone H3 lysine 9 (H3K9) trimethylase, *Kmt1a*. Interestingly, they did not detect global changes in the levels of H3K9me3 by western blot. The authors suggested that subtle changes may have occurred [73].

### 2.3. Maternal Obesity Postnatally: Effects in the Breast Milk

Breast milk carries a myriad of nutrients and molecules, such as antibodies [49], microRNA (miRNAs) [74], and antioxidants [75]. It feeds and provides immunological defenses for the growing baby. The maternal diet and obesity can influence the lipid content [76,77], the protein levels [78], and the levels of some miRNAs and other non-coding RNA (ncRNAs) [79] in breast milk.

Early postnatal life exposures can influence the development of a metabolic phenotype, which is believed to be mediated through breast milk [49,80]. The importance of breast milk’s effects has been demonstrated by Gorski et al. [81]. Their study showed that obesity-prone rat pups that were cross-fostered with lean mothers during suckling ameliorated their insulin resistance as adults. On the other hand, obesity-resistant pups that were cross-fostered with obese mothers showed reduced insulin sensitivity and had a diet-induced increase in adiposity. These effects were possibly associated with changes in hypothalamic neuropeptides, such as insulin and leptin receptors [81]. Moreover, in rats, a maternal HFD during suckling can program visceral adiposity and the epigenetic regulation of epididymal white adipose tissue (eWAT). In that study, the male offspring of HFD-fed mothers presented an increment in eWAT that was associated with higher levels of stearoyl-CoA desaturase-1 (*Scd1*), coupled with a decrease in methylation levels in its promoter [80]. *Scd1* is a key enzyme of fatty acid (FA) metabolism that converts saturated FAs to monounsaturated FAs, which are the predominant substrates for triglyceride synthesis [80]. Importantly, the resulting increment in FA content in breast milk prompted the authors to propose them as metabolic mediators.

Overall, these studies show that breast milk from HFD-fed or obese mothers affect offspring health. However, the mechanism is not clear. It is possible that breast milk components could be involved. More research is required to determine whether it is the fat itself, ncRNAs, their combination, or something else that is involved in the mechanism.

### 2.4. Maternal Effects: What Is Known and What Is Missing

**Figure 1:**
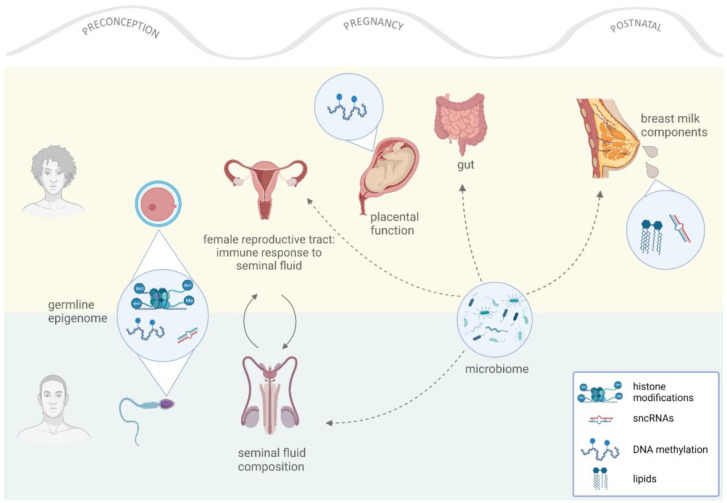
Effects of parental nutrition in offspring. Both parents contribute to their offspring’s health in different ways. The maternal contribution ranges from conception until postnatal care, while the paternal contribution occurs at conception. Different mediators (for example, germ cells) play roles during different developmental windows, transferring “environmental information” to offspring through epigenetic and/or non-genetic mechanisms.

There are three developmental windows of susceptibility for a HFD. This environmental challenge induces epigenetic alterations in the oocyte, the placenta, and, putatively, in breast milk composition, thereby impacting the next generation (Figure 1 and Table 1). However, further studies are required to fully elucidate the mechanistic details of how such epigenetic information is encoded during the different susceptibility time windows and how it is inherited by offspring. It is also important to determine whether one of these windows confers more susceptibility than the others. It is still not clear if epigenetic signals are reversible and how they are maintained during F1 development and adulthood.

## 3. Paternal Obesity Affects the Offspring Health

Unlike mothers, fathers have one short time window within which to pass their “environmental memory” to their offspring: at conception. Originally, this served as one of the reasons for underestimating the paternal contribution to offspring health. Global reprogramming of the sperm epigenome in early embryogenesis and transcriptional silencing during sperm maturation have rendered paternal intergenerational inheritance less feasible in affecting offspring health [82,83,84]. However, seminal studies of various environmental influences strongly suggest the opposite [85,86,87,88]. Paternal exposure to a HFD has been shown to induce a broad range of inter- and transgenerational negative effects, including programming of β-cell dysfunction; dysregulation of hepatic genes; cognitive, metabolic, and reproductive impairments; and female predisposition to breast cancer [87,89].

With advancements in research, several key findings have increased interest in the epigenetic inheritance of environmental effects via male germ cells. On the one hand, although most of the histones are gradually replaced with protamines during mammalian spermatogenesis, up to 8% and 15% of nucleosomes are retained in mice and humans, respectively [90]. Enriched for the genes involved in embryonic development, these escaped regions tend to be a plausible candidate for paternal epigenetic inheritance [91,92]. On the other hand, the incomplete erasure of paternal methylome in early embryogenesis on intergenic and protein-coding regions also applies to genes that are involved in neuronal development [93]. Moreover, imprinted regions escape the first wave of global demethylation; therefore, they remain methylated in the somatic cells of offspring. Despite comprising ~1% of the human genes [94], some portion of environmentally acquired epigenetic marks could be transferred via those parent-of-origin-specific signatures. Thus, differentially methylated regions (DMRs) of paternal origin, within or outside imprinted regions, can evade reprogramming during early embryogenesis. In addition, emerging evidence supports the role of transcription factors as carriers of epigenetic information during spermatogenesis and pre-implantation development, defining the methylation status of paternal DNA [95]. Finally, although transcriptionally inert, mature spermatozoa is rich in small non-coding RNAs (sncRNAs), another putative epifactor that could reflect and pass along environmental influences through generations [96].

Interestingly, in addition to germline effects, seminal fluid components and microbiota have also been implicated in intergenerational inheritance [55,97]. These additional layers provide further support for epidemiological studies that reveal the extent and mechanistic complexity of the paternal contribution to offspring health (Figure 1).

### 3.1. Paternal Obesity at Conception: Effects in Sperm

The original study of intergenerational non-genetic effects in a HFD rat model addressed whether excessive fat consumption could induce changes in methylation and gene expression levels in pancreatic islets [87]. Whether metabolic effects can also be established in sperm and, therefore, be transmitted to the following generation has been addressed in multiple studies since then (Table 2). As discussed in the following sections, the current research is focused on DNA methylation, histone modifications, and sncRNAs, the latter being the most broadly researched and most likely mediator of paternal effects.

#### 3.1.1. DNA Methylation

Pioneering rodent studies examining changes in male germ cells revealed that the methylome of spermatozoa is sensitive to a HFD. For example, a chronic HFD caused global hypomethylation in mouse sperm [98] and resulted in DMRs in spermatozoa from F0 as well as F1 rats, with the latter being unexposed to dietary challenge [99]. However, whether these changes are essential and/or sufficient for transmitting metabolic phenotypes through generations remains subject to debate. In fact, although there were some common DMRs detected in the founder and offspring sperm, gene regulation associated with established methylation signatures did not persist in the affected somatic tissues across generations [99]. Such inconsistencies have also been reported in other rodent studies [88,100] and have been, to a certain degree, ascribed to technical bias from methylome experiments [91,101]. Moreover, a study closely examining genome-wide methylation in the sperm suggested that most of the signal originates from genetic or epigenetic variations that are not linked to a HFD [101]. Despite these discrepancies, differential methylation remains one of the broadly addressed readouts for studying the transmission of HFD effects. An interesting hypothesis prevails in the field; it established that modest changes in sperm methylome may modulate HFD-induced changes at early stages of developmental programming, which can manifest as a metabolic dysfunction later in life.

Several human studies have also evidenced DMRs in the sperm of obese individuals versus lean individuals. For example, Donkin et al. identified DMRs in the proximity of genes responsible for central nervous system development and metabolic processes [102]. Consistently, differential methylation within and outside paternally imprinted loci have been recently reported in gametes of overweight and obese men and were further associated with unhealthy diets rich in fat [103,104,105,106].

Although direct testing of the transmission of such changes intergenerationally in humans is ethically limited, paternal obesity has been shown to be associated with offspring methylation status in the cord blood in paternally imprinted genes, indicating a possible transmission of such signatures via sperm [107]. Interestingly, a similar approach has been attempted in a larger cohort undergoing in vitro fertilization or intracytoplasmic sperm injection. That study revealed an association between differential methylation levels in some paternally imprinted regions in the sperm of obese donors and the cord blood of their babies [104].

#### 3.1.2. Histone Modification

Obesity-related impairments during spermatogenesis and its possible mediation via altered histone modifications have been reported by multiple research groups [108,109,110,111]. However, the transmission of altered histone epimutations to the following generation remains unresolved.

HFD-induced differential profiles of histone post-translational modifications have been identified in a few studies. For instance, Terashima et al. revealed a differential occupancy of histone H3 in genes involved in early embryonic development in HFD-fed mouse sperm [108]. More recently, decreased H3K9 dimethylation was also reported in a similar experimental setting [109]. However, the transfer of altered sperm histone signatures to the embryo is challenged by the current understanding of early developmental processes, in which sperm histone marks become heavily reprogrammed [112]. Regardless, findings from transgenic mouse experiments strongly support the critical role of sperm histones in embryonic development and, importantly, reveal the propagation of the resulting phenotype transgenerationally [110]. In fact, germline-specific overexpression of lysine demethylase KDM1A and the resulting reduced H3K4 methylation levels lead to reduced survival and developmental defects spanning multiple generations that originate from heterozygous breeding. Interestingly, a recent follow-up study combined the mentioned transgenic effect with the HFD challenge. The results suggested that altered H3K4 trimethylation levels correspond to embryonic transcription and chromatin profiles in HFD-fed wild-type (WT) mice as well as in transgenic mice, with the latter resulting in a stronger metabolic phenotype transmitted transgenerationally [111].

Overall, although undetected in a human study [102], increasing evidence from experimental models suggests that sperm-derived histones can sense and possibly transfer information on the paternal metabolic state. A precise mechanistic model, however, remains to be elucidated.

#### 3.1.3. sncRNAs

Several rodent studies have detected alterations of sperm-derived sncRNAs levels as a consequence of an obesogenic diet. For example, a HFD and a WD induced differential expression of miRNAs, tRNA-derived fragments (tRFs), and piwi-interacting RNAs (piRNAs); upon their subsequent injection into the normal oocyte or one-cell embryo, a compromised metabolic phenotype resulted in offspring [98,113,114]. Interestingly, some researchers examined individual biotypes (e.g., miRNAs and tRFs) or a specific subtype to define their significance and sufficiency in establishing a metabolic phenotype. For instance, Grandjean et al. focused on the most differentially expressed miR19b (microRNA 19b) in WD mouse sperm. This single miRNA subtype led to partial penetrance of metabolic alterations [114]. In another study, a total tRF pool resulted in efficient mimicking of total sperm RNA-induced phenotypes [113].

Further supported by human studies, sncRNAs appear to sense diet-induced changes in a metabolic environment and to transmit the signature to the following generations [91,102]. The pioneering human study comparing sncRNA expression profiles in obese and normal individuals revealed differential abundance of piRNAs with putative targets involved in neurogenesis. Interestingly, a reanalysis of the sncRNA expression data with a more fine-tuned pipeline revealed that a set of tRFs were also differentially expressed between the two groups, suggesting their role as the carrier of “environmental information” in human spermatozoa [115]. An increasing number of findings that support the contribution of tRFs has followed the discovery of the essential role of RNA modifications. In fact, post-transcriptional methylation of tRFs proved to be critical in transmitting the HFD-induced phenotype intergenerationally [113,116].

sncRNAs have been recently implicated in multigenerational effects resulting from ancestral diet-induced obesity [117]. Exposing male mice to WD for five consecutive generations resulted in metabolic phenotype exacerbating across downstream generations. Interestingly, sncRNAs were differentially expressed in F1 but not in F5. To further investigate the role of sncRNAs in inter- or transgenerational metabolic effects, the total RNAs from F1 and F5 obese mice were microinjected into the naïve zygotes and the resulting effects were compared to those of natural matings. While the metabolic phenotype persisted through the following two generations, it did not aggravate along the generations, as was observed with natural matings. Altogether, these findings indicate the role of sncRNAs in the relatively short-term paternal inheritance of diet-induced obesity and related phenotypes, whereas long-term penetrance of the effects points at a more complex mechanistic level, such as cooperation between epigenetic factors.

Overall, while rapidly advancing, the research requires better tools to identify the origin of sncRNAs and to trace their transmission and effects at the site of action during embryonic or postnatal development. Although microinjection can be a robust tool to ascribe altered phenotypes to sperm head content, it does not enable us to detect when, where, and how the effects are established. Therefore, several key aspects remain to be addressed. On the one hand, due to sncRNA dilution throughout mammalian embryonic cleavages, the information they carry could only be communicated at an early stage of development. Alternatively, sncRNAs transferred from sperm to embryos may activate a self-amplifying mechanism to overcome this caveat and persist throughout development. In turn, it has been shown that the offspring of HFD males exert small RNA profiles in the adipose tissue, similar to the founder sperm [99]. Considering the observed acquisition of small RNAs from epididymis by sperm during its maturation, the overall picture becomes even more complex [118]. Therefore, distinguishing the sperm-born sncRNAs from those obtained through soma-to-germline transition and exploring their trajectory throughout the different stages of offspring development are some primary requirements to eliminate discrepancies that are related to the paternal epigenetic inheritance of diet-induced obesity. As an example, a recently developed tool for the efficient tagging and tracking of sncRNAs in vivo can help address the mentioned aspects in future studies [119].

## 4. Beyond Epigenome

We have provided an overview of the intergenerational consequences of parental obesity that are induced or mediated bona fide by epigenetic mechanisms. Lately, studies have highlighted an equally important role of parental modifiers of offspring health beyond the epigenome (for a review, see [120,121]). In the following section, we briefly review the known facts about the relevance of the oocyte environment, the seminal fluid, and the parental microbiota composition in the intergenerational transmission of non-genetic information and phenotype programming (Figure 1). This is an emerging part of the epigenetic inheritance field; the obvious question on whether these reported phenomena are really non-epigenetic or, rather, signaling mediators to the parental epigenomes is yet to be answered.

### 4.1. Oocyte Environment

Oocyte quality is essential for female fertility and offspring health, and the developmental environment of the maturing oocyte is essential for optimal oocyte quality. An obesogenic diet, for example, has been shown to alter the reproductive function and offspring health by changing the biochemical environment of the follicle and inducing endoplasmic reticulum stress, telomere dysfunction [122], mitochondrial dysfunction, oxidative stress, and lipid droplet accumulation [123].

### 4.2. Seminal Plasma and the Maternal Reproductive Tract Immune Response

The seminal plasma is the fluid that contains the secretions from the male accessory glands (i.e., the seminal vesicle and the prostate and bulbourethral glands), the testes, and the epididymis. Until recently, the seminal plasma has been seen merely as the medium to transport and support the sperm into the maternal reproductive tract. Recent studies—pioneered by the research group of Sarah Robertson et al.—have shown that the composition of the seminal plasma is important in signaling the female tract at conception by priming a tolerogenic response that is essential for embryo implantation, remodeling of the uterine blood vessels, and proper placentation [124,125,126,127]. The depletion of uterine regulatory T-cells (the main mediators of immune tolerance) results in implantation failure, fetal loss, or fetal growth restriction in mice [128,129,130].

Interestingly, the composition of the seminal plasma is affected by lifestyle factors. For example, exposure to an obesogenic diet changes the composition of the seminal plasma toward a pro-inflammatory cytokine repertoire, which alters female immune adaptation to pregnancy and ultimately affects pregnancy and offspring health [55,131,132,133]. Furthermore, we have recently shown that paternal circadian disruption at conception reprograms offspring metabolism by affecting the rhythmicity of corticosterone secretion into the seminal plasma and inducing placental dysfunction and fetal growth restriction [134].

Altogether, these findings suggest that the seminal plasma, although not essential for fertilization and pregnancy establishment, is important for pregnancy outcomes, including embryo implantation, placentation, post-fertilization embryonic development, and fetal growth, and ultimately affects adult offspring health [135].

### 4.3. Microbiota

Mammalian bodies are colonized by microbes, which respond to the environment mainly by altering the circulating metabolome and, thereby, influencing host physiology, health, and disease trajectories (for a review, see [136]). The most important feature of the microbiota is its ability to transfer phenotypes across organisms, which has recently boosted microbiota transplantation as a therapeutic tool for non-communicable diseases, including diabetes, obesity, and chronic inflammatory disorders (for a review, see [137]).

Of utmost importance for this review, microbiota—and in particular those of maternal origin—are of critical importance in determining offspring health [138]. The alteration of microbiota during pregnancy affects the development and adult phenotypes in mice [138,139,140,141,142] and is one of the main causes of adverse pregnancy outcomes affecting neonatal and infant health in humans [143,144,145,146].

In addition to the indirect effects of the maternal microbiome during pregnancy, there is experimental and clinical evidence showing that the maternal microbiome is present in the breast milk [147] and in the vaginal tract [143]; from these sites, they are directly transferred to the offspring gut [147,148]. Breast milk microbiota composition is modified by the maternal diet and by obesity in mice and humans [50,51,52,53,149] and has been reported as affecting offspring development and health [149]. Likewise, the composition of the vaginal environment, including microbiome, is modified by the maternal diet [150,151] and is critical for offspring metabolic and neurobehavioral phenotypes [152,153,154].

Less is known about the effects of the paternal microbiota. Two studies from the same research group reported the presence of a microbiome in the mouse seminal plasma and its responsiveness to a HFD treatment [97,155]. Although largely unexplored, there is ongoing discussion in the scientific community about the potential role of the seminal microbiome for offspring health, most likely through immune modulation in the female tract at conception [156].

Although the highlighted biological features of the microbiome make it a potential novel modulator of acquired inheritance, the available evidence in mammals is still too uncertain to support conclusions about a role for the microbiota in the parental programming of offspring phenotypes.

## 5. Reversibility

To what extent can the effects of parental obesity for offspring health be reversed? Having demonstrated the legacy of parental and offspring health in mammals, the epigenetic inheritance field is focusing on the prevention or reversal of this legacy. The vast majority of studies aimed at rescuing offspring health by re-establishing parental health preconceptionally and/or during gestation. The three most often reported approaches for parental obesity (summarized below) are dietary intervention, exercise, and bariatric surgery. In addition, other studies that are not reported below have tested probiotics and dietary supplements [157,158,159].

### 5.1. Lifestyle Interventions: Diet and Exercise

Lifestyle interventions, including diet, exercise, and their combination, are the most commonly used approaches, in humans and model organisms, to reverse common obesity. The scientific community has applied lifestyle interventions to test the reversibility of intergenerational obesity in several mammalian model organisms.

Studies of mice have shown that the correction of maternal obesity with a diet that is poor in fat may improve the metabolism in offspring who are on a control diet [160,161,162,163,164,165], while worsening their susceptibility to diet-induced obesity when the change of diet is short- or medium-term before pregnancy [161,162,163,166]. Maternal exercise before and during pregnancy has been proven to be efficient in reducing the degree of liver damage in offspring of obese female mice [167], while a combination of diet and exercise improved placental vascularization and prevented macrosomia in offspring of obese mice mothers [168]. These findings have been further supported by independent studies of rats [21] or Japanese macaques [169], showing that correction of maternal obesity before pregnancy partially improves the overall health of offspring when they are raised on a standard diet.

Interestingly, the same lifestyle interventions appear to be effective for the intergenerational consequences of paternal obesity. Studies of mice have the reported beneficial effects of paternal dietary intervention for the metabolic and reproductive health of offspring [170,171]. Likewise, preconceptional exercise, alone or in combination with a dietary intervention, improved metabolic health in obese male mice and positively affected offspring embryonic development [172], adult metabolic health (in particular, body composition and lipid homeostasis) [173], and reproductive health [170,174].

### 5.2. Bariatric Surgery

In addition to common lifestyle interventions, the more severe forms of obesity are now treated by bariatric surgery, which is becoming increasingly common. Reported evidence based on studies of humans suggests that bariatric surgery is associated with both positive and negative pregnancy and neonatal outcomes [39,175,176]. These findings have been corroborated by studies in diet-induced obesity rats, where ventral sleeve gastrectomy has led to worsened pregnancies and offspring health [177,178,179]. Not many studies have been reported on paternal preconceptional bariatric surgery, but an interesting recent report revealed DMRs in spermatozoa before and after gastric bypass surgery at time points of one week and one year, suggesting the potential of bariatric surgery to modify the sperm epigenome and, therefore, potentially have paternal intergenerational consequences [102].

The evidence on the benefits of weight loss via surgery before pregnancy is still contradictory or unclear. The effects may depend on the initial maternal BMI, the weight reduction, the surgical procedure chosen, the metabolic effects of the surgery, and the age of the offspring when comparing the data [180]. Given that a change in diet long before pregnancy has positive effects in offspring, it is possible that women should wait after surgery before becoming pregnant and establish a healthy diet following the surgical procedure. Although it is recommended that women wait 12 to 18 months after the surgery, there is no clear data supporting the benefit of such a wait [39,175].

### 5.3. Reversibility: Chances of Success

Contrary to genetic mutations, epigenetic reprogramming is, by definition, reversible. Therefore, modifying one’s lifestyle and restoring parental health before conception or during pregnancy has the potential to mitigate the effects of parental obesity and its consequences on offspring health.

While reporting interesting results, available studies, including the ones mentioned in this review, provide a non-conclusive patchwork of different intervention methods and lengths, exposure windows, dietary combinations, and phenotypic analyses that would need to be harmonized by more robust experimental designs, independent cross-validation studies, and aligned phenotyping pipelines to deliver useful results for the wider scientific and clinical communities. This assumes that parental epigenome reprogramming is secondary to a certain phenotypic manifestation rather than, for example, a specific environmental challenge.

## 6. Concluding Remarks: When and How Can a Parent Affect Offspring Health?

The central OneGenotype–OnePhenotype dogma was rejected many years ago by the observation of significant phenotypic plasticity in isogenic individuals, mostly due to gene–gene and gene–environment interactions. The discovery of epigenetic inheritance, more than a decade ago, has further highlighted the observation that individual phenotypes are not only determined by one’s own genes and gene–environment interaction, but also by parental effects through epigenetic mechanisms. This hypothesis is particularly fascinating in the case of non-communicable diseases, such as obesity and diabetes, whose pathogenesis is significantly contributed to by gene–environment interactions and, therefore, by lifestyle choices.

Here, we provided an overview of the state-of-the-art view of the role of epigenetic inheritance and parental effects in the rapid worldwide spread of obesity and associated comorbidities. Although compelling evidence supports the relevance of parental metabolic health at conception or during gestation for offspring metabolic homeostasis and susceptibility to metabolic diseases, the field still lacks a comprehensive understanding of the underlying molecular mechanisms. Indeed, both germ-cell and non-germ cell factors have been implicated in such parental effects.

We further touched upon the possibility of reversing parental effects by restoring parental health via lifestyle intervention and bariatric surgery. Although interesting, this set of findings is still far from being conclusive and, as highlighted, needs better and more substantial experimental and clinical evidence.

Where do we stand? We have unearthed an interesting and fascinating realm of knowledge, which surely holds secrets that are fundamental to our understanding of human physiology and pathophysiology, and which is useful for the management and prevention of diseases—such as obesity and diabetes—that are spreading like wildfire, especially among the younger generation. Further effort by the entire scientific community is required. Greater international cooperation, cross-validation, harmonization of protocols, constructive feedback, and exchanges of ideas are suggested ways of transforming epigenetic inheritance and parental programming from fascinating ideas into a useful reality.

## Figures and Tables

**Table 1:** Putative epigenetic mechanisms that are associated with the intergenerational consequences of maternal obesity.

Diet	Length/Period When Consumed	Species	Suggested Mechanism of Transmission	References
HFD—45% fat (MD12032)	12 weeks	mouse	global decrease in 5mC in oocytes	Hou et al., 2016 [59]
HFD—60% fat (H10060)	eight weeks	mouse	global decrease of 5mC and H3K4me3; global increase of 5mhC and H3K9me3 in oocytes	Huang et al., 2022 [60]
HFD—60 kcal% fat (D12492)	12 weeks	mouse	increased DNA methylation in leptin promoter and altered DNA methylation in the peroxisome proliferator-activated receptor alpha (*Pparα*) promoter	Ge et al., 2014 [61]
HFD—60 kcal% fat (D12492)	15 days after mating	mouse	global hypomethylation in female placentas	Gallou-Kabani et al., 2010 [68]
maternal BMI was assessed before week 10 of gestation and maternal diet composition was assessed using 3-day food records at each trimester	NA (not applicable)	human	altered DNA methylation of genes related to several biological processes (SREBP signaling, chromatin remodeling, IGF receptor signaling, PI3-kinase signaling, and nitric oxide synthase)	Thakali et al., 2020 [71]
HFD—60 kcal% fat (D12492)	15 days after mating	mouse	downregulation of the H3K9 trimethylase (*Kmt1a*) without global changes in levels of the H3K9me3 by western blot	Gabory et al., 2012 [73]
HFD—60% kcal fat (D12492)	entire lactation period	rats	decrease in methylation levels in *Scd1* promoter coupled with higher expression levels of the gene	Butruille et al., 2019 [80]
high-energy (HE) 4.41 kcal/g—32% as fat (D12266B)	three months before mating, through gestation, and weaning	rats	changes in hypothalamic neuropeptides, such as insulin and leptin receptors	Gorski et al., 2006 [81]

The majority of the studies evaluated metabolic phenotypes in offspring.

**Table 2:** Putative epigenetic mechanisms associated with the intergenerational consequences of paternal obesity.

Diet	Length/Period When It Is Consumed	Species	Suggested Mechanism of Transmission	References
HFD—21% fat (TD.88137)	10 weeks before mating	mouse	hypomethylation sperm DNA; differentially expressed sperm miRNA	Fullston et al., 2013 [98] *
HFD—42/45% fat (TD.88137/TD.08811)	12 weeks before mating	rat	altered methylation in DMRs; differentially expressed miRNA in sperm	de Castro Barbosa et al., 2015 [99] *
obesity (BMI > 29.7, median BMI of 31.8)and glucose intolerance	NA	human	altered methylation in DMRs; differentially expressed sncRNA in sperm	Donkin et al., 2016 [102]
obesity (BMI > 25)	NA	human	altered methylation in DMRs in mature spermatozoa	Keyhan et al., 2021 [103]
underweight (BMI < 19), normal weight (BMI: 19–24.9), pre-obesity (BMI: 25–29.9), obesity (BMI: 30–40.3)	NA	human	increased methylation in maternally expressed gene 3 intergenic (*MEG3*-IG) DMR in sperm	Potabattula et al., 2019 [104]
overweight (25 ≤ BMI < 30)/obesity (BMI ≥ 30)	NA	human	altered methylation in certain imprinted genes DMRs in sperm	Soubry et al., 2016 [105]
unhealthy (“fast”) food	record of food intake from the last seven days before the study	human	increased methylation in DMRs of certain imprinted genes in sperm	Soubry et al., 2021 [106]
HFD—60% fat	10 weeks before mating	mouse	differential H3 retention in spermatozoa	Terashima, M. et al., 2015 [108]
HFD—45% fat	three months before mating	mouse	decreased H3K9 dimethylation in spermatozoa	Claycombe-Larson et al., 2020 [109]
HFD—60% fat (D12492)WT or transgenic overexpressing KDM1A	10–12 weeks before mating	mouse	alterations in H3K4me3 in spermatozoa	Pepin et al., 2022 [111] *
HFD—60% fat	six months before fertilization	mouse	differentially expressed sperm-derived sncRNAs and their modifications	Chen et al., 2016 [113]
WD—21% fat, 34% sucrose (Western 1635, SAFE)	four months before mating	mouse	sperm or testis-derived total RNA; differentially expressed individual miRNAs	Grandjean et al., 2015 [114]
HFD—60% fat (D12492)WT or *Dnmt2* knockout	from six weeks to six months of age	mouse	differentially expressed sperm-derived sncRNAs and their modifications	Zhang et al., 2018 [116]
WD—45% fat (U8954 version 205)	multigenerational: three months before mating, repeated for five consecutive generations	mouse	differentially expressed sperm-derived sncRNAs for partial transmission of metabolic phenotype	Raad et al., 2021 [117] *

The majority of the studies evaluated metabolic phenotypes in the offspring. * Transgenerational phenotype(s) were also evaluated.

## Data Availability

Not applicable.

## Acronyms

5hmC	5-hydroxymethylcytosine
5mC	5-methylcytosine
BMI	body mass index
DMRs	differentially methylated regions
DOHaD	developmental origins of health and disease
eWAT	epididymal white adipose tissue
FA	fatty acid
*FTO*	fat mass and obesity associated gene
GWAS	genome wide association studies
H3K4	histone H3 lysine 4
H3K4me3	histone H3 lysine 4 trimethylation
H3K9	histone H3 lysine 9
H3K9me3	histone H3 lysine 9 trimethylation
HE	high energy
HFD	high-fat diet
IGF	insulin-like growth factor
*Igf2*	insulin-like growth factor 2
KDM1A	lysine demethylase 1A
*Kmt1a*	H3K9 trimethylase
*MC4R*	melanocortin 4 receptor gene
*MEG3*-IG	maternally expressed gene 3 intergenic
miR19b	microRNA 19b
miRNA	microRNA
NA	not applicable
ncRNA	non-coding RNA
PI3-kinase	phosphoinositid-3-kinase
piRNA	piwi-interacting RNA
POHaD	paternal origin of health and disease
*Pparα*	peroxisome proliferator-activated receptor alpha
*Scd1*	stearoyl-CoA desaturase-1
sncRNA	small non-coding RNA
SREBP	sterol regulatory element-binding protein
T2D	type 2 diabetes
tRFs	tRNA-derived fragments
WD	Western diet
WT	wild-type
